# Long-Term Overconsumption of Sugar Starting at Adolescence Produces Persistent Hyperactivity and Neurocognitive Deficits in Adulthood

**DOI:** 10.3389/fnins.2021.670430

**Published:** 2021-06-07

**Authors:** Kate Beecher, Ignatius Alvarez Cooper, Joshua Wang, Shaun B. Walters, Fatemeh Chehrehasa, Selena E. Bartlett, Arnauld Belmer

**Affiliations:** ^1^Addiction Neuroscience and Obesity Laboratory, School of Clinical Sciences, Translational Research Institute, Faculty of Health, Queensland University of Technology, Brisbane, QLD, Australia; ^2^Addiction Neuroscience and Obesity Laboratory, School of Biomedical Sciences, Translational Research Institute, Faculty of Health, Queensland University of Technology, Brisbane, QLD, Australia; ^3^School of Biomedical Sciences, University of Queensland, Brisbane, QLD, Australia

**Keywords:** sucrose, hyperactivity, neurocognitive deficits, neurogenesis, adulthood

## Abstract

Sugar has become embedded in modern food and beverages. This has led to overconsumption of sugar in children, adolescents, and adults, with more than 60 countries consuming more than four times (>100 g/person/day) the WHO recommendations (25 g/person/day). Recent evidence suggests that obesity and impulsivity from poor dietary habits leads to further overconsumption of processed food and beverages. The long-term effects on cognitive processes and hyperactivity from sugar overconsumption, beginning at adolescence are not known. Using a well-validated mouse model of sugar consumption, we found that long-term sugar consumption, at a level that significantly augments weight gain, elicits an abnormal hyperlocomotor response to novelty and alters both episodic and spatial memory. Our results are similar to those reported in attention deficit and hyperactivity disorders. The deficits in hippocampal-dependent learning and memory were accompanied by altered hippocampal neurogenesis, with an overall decrease in the proliferation and differentiation of newborn neurons within the dentate gyrus. This suggests that long-term overconsumption of sugar, as that which occurs in the Western Diet might contribute to an increased risk of developing persistent hyperactivity and neurocognitive deficits in adulthood.

## Introduction

The concept of “sugar addiction” and the classification of sugar as a substance of abuse are still debated. There is, however, increasing evidence of overlap in the brain circuitry and molecular signaling pathways involved in sugar consumption and drug abuse (for recent review see Jacques et al., 2019). Humans consume sugar and food to regulate homeostatic energy balance, but also for pleasure and comfort. This hedonistic desire for palatable food is reward-driven and overeating may result in maladaptive/negative neuroplasticity that overrides homeostatic regulation (Kenny, 2011). In humans, sugar and sweetness can induce dopamine release, reward and craving that are comparable in magnitude to those induced by addictive drugs, suggesting that sugar changes brain reward signaling and circuitry similar to other drugs of abuse (Avena and Hoebel, 2003b; Rada et al., 2005; Lenoir et al., 2007; Klenowski et al., 2016; Shariff et al., 2016, 2017).

High sugar and/or high fat diets have been shown to precipitate addiction-like psychiatric phenotypes in a number of rodent studies (Avena et al., 2009; Avena, 2010; Criscitelli and Avena, 2016). In rats, intermittent consumption of 10% (w/v) sucrose or 25% (w/v) glucose solution) elicits hallmark signs of addictive behavior such as binging, tolerance, craving (Rada et al., 2005), cross-sensitization (Avena and Hoebel, 2003b) and symptoms of withdrawal (Colantuoni et al., 2002; Avena et al., 2008) such as anxiety-(Colantuoni et al., 2002; Avena et al., 2008; Parylak et al., 2012; Eudave et al., 2018; Gueye et al., 2018; Xu and Reichelt, 2018) and depressive-like behaviors (Vollmayr et al., 2004; Iemolo et al., 2012; Harrell et al., 2015; Santos et al., 2018). In addition, sugar consumption has been shown to increase reward seeking, impulsivity to feed and compulsivity in rats willing to endure noxious stimuli such as extreme cold, heat and foot-shock to procure sugar and highly palatable foods (Cabanac and Johnson, 1983; Avena et al., 2005; Foo and Mason, 2005; Oswald et al., 2011). Interestingly, rats are also more resilient to foot shock punishments when seeking for palatable food compared to methamphetamine (Krasnova et al., 2014).

Increasing evidence shows that unrestricted consumption of high-sugar food and beverages within the Western Diet might be linked to the increased obesity epidemic (Stanhope, 2016; Johnson et al., 2017; Freeman et al., 2018; Yoshida and Simoes, 2018; Sigala and Stanhope, 2021). A strong association between attention-deficits/hyperactivity disorders (ADHD) and overweight/obesity have further been revealed (Altfas, 2002; Strimas et al., 2008; Cortese et al., 2016; Cortese, 2019). Taken together, these data suggest that sugar-induced obesity may participate to the developing pathogenesis of ADHD-like symptoms in western countries. In children, high sugar consumption correlates with hyperactivity (Kim and Chang, 2011) and in adults, with inattention and impulsivity (Li et al., 2020). However, some inconsistencies remain regarding the potential correlation (Yu et al., 2016; Farsad-Naeimi et al., 2020) or not (Del-Ponte et al., 2019) with ADHD (Johnson et al., 2011; Paglia, 2019). In rodents, high-sucrose consumption also impairs neurocognitive functions such as spatial learning, object recognition, behavioral inhibition and fear-memory (Kendig, 2014; Reichelt et al., 2015; Kruse et al., 2019; Spoelma and Boakes, 2021). Interestingly, high sucrose intake during pregnancy elicits ADHD-like behavioral phenotypes in mice offspring, with increased locomotor activity, reduced attention/learning and impulsivity (Choi et al., 2015). Anxiety, depression, and cognitive deficits are strongly associated with impaired hippocampal neurogenesis in animal models, although evidence for a causative relationship is often lacking. Indeed, anxiety and spatial memory deficits elicited by long-term consumption of sucrose are accompanied by alterations in hippocampal neurogenesis and physiology (Molteni et al., 2002; Stranahan et al., 2008; van der Borght et al., 2011; Lemos et al., 2016; Reichelt et al., 2016). While drugs of abuse such as ethanol are known to negatively affect neurogenesis, the effect of high levels of sugar consumption requires further characterization since link between neurogenesis to anxiety and depression has not been fully explored (Xu and Reichelt, 2018).

Sucrose became embedded in modern food and beverages, and the aforementioned studies suggest that sugar overconsumption satisfies all criteria for the classification of sugar as a drug of abuse, with its chronic abuse proposed to produce overweight, locomotor, emotional and cognitive impairments. However, it remains unclear whether a lifetime of chronic overconsumption of sucrose, starting at adolescence, affects locomotor behavior, emotions and cognition through adulthood. Therefore, we used a mouse model of long-term intake of sucrose to determine the effects on locomotion, anxiety, memory, and hippocampal neurogenesis. Our results show for the first time that long-term consumption of sucrose leads to significant weight gain and produces persistent hyperactivity and learning impairments, correlated to reduced hippocampal neurogenesis in adult mice. These results suggest that long-term sugar intake in the Western Diet might play a role in the pathogenesis of attention deficits and hyperactivity-related disorders.

## Materials and Methods

### Animals and Housing

Five-week-old male C57BL/6J mice (ARC, WA, Australia) were individually housed under reverse-light cycle conditions (lights off at 9:00 am) in a climate-controlled room with *ad libitum* access to food (standard mouse chow) and water. Following one week of habituation to the housing conditions, mice were offered sucrose or water during the drinking sessions. All procedures were approved by The University of Queensland and The Queensland University of Technology Animal Ethics Committees under approval QUT/053/18 and complied with the policies and regulations regarding animal experimentation and other ethical matters, in accordance with the Queensland Government Animal Research Act 2001, associated Animal Care and Protection Regulations (2002 and 2008), as well as the Australian Code for the Care and Use of Animals for Scientific Purposes, 8th Edition (National Health and Medical Research Council, 2013).

### Sucrose Consumption

All mice had food and acidified-filtered water available at all times. The sucrose solution was freshly prepared weekly and was presented in 50 ml plastic falcon tubes fitted with rubber stoppers and a 6.35 cm stainless-steel sipper tube with double ball bearings. Mouse weights were measured daily to calculate the adjusted g/kg intake of sucrose. Two groups of mice (*n* = 23 mice/group) had *ad libitum* access to 25% sucrose or water for 12 weeks. Briefly, mice were given access to one bottle of 25% (w/v) sucrose solution and one bottle of water available at all times, or two bottles of water (controls) available at all times. Sucrose and water containing bottles were weighed daily. Two other groups of mice (*n* = 23 mice/group) were trained in the in a restricted access model of sugar consumption (Drinking-In-the-Dark) for 12 weeks as previously described (Rhodes et al., 2005; Patkar et al., 2017, 2019; Belmer et al., 2018). Briefly, mice were given access to one bottle of 25% (w/v) sucrose for a 2 h period Monday to Friday. Drinking sessions started 3 h into the dark cycle. Sucrose containing bottles were weighed prior to presentation, as well as 2 h after presentation.

### Behavioral Testing

**FIGURE 1:**
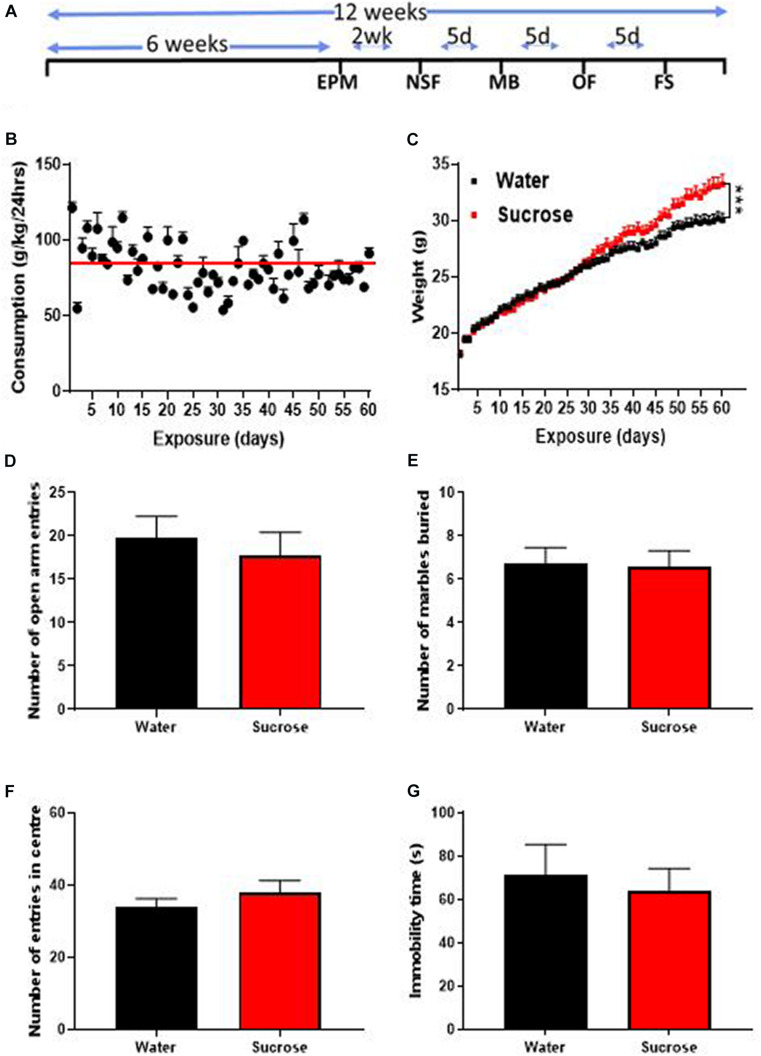
Long-term sucrose consumption increases mouse weights without altering emotional behavior. **(A)** Experimental design of the anxiety-related, depression-related, general locomotor activity and impulse control behavioral testing in elevated-plus-maze (EPM), novelty suppressed feeding (NSF), marble burying (MB), open field (OF) and forced swimming test (FS). Animals consumed 25% sucrose for 5 weeks prior to each behavioral testing and continued to be exposed to sucrose for a total of 12 weeks. Behavioral tests were initially conducted 2 weeks (2 wk) then continued every 5 days (5d) after 24 h of sugar withdrawal. Animals were assigned into two groups: sugar-withdrawn animals and water control. **(B)** Mice exhibit stable levels of long-term sucrose intake of 82.2 ± 0.9 g/kg indicated by the red line. **(C)** There was a significant difference in the weights of animals consuming sucrose, compared to water control, starting after 4 weeks, and continuing throughout the 12 weeks. Data are presented as mean ± SEM; *n* = 8 mice/group, *t*-test, ***p < 0.001. **(D–F)** Long-term sucrose consumption does not alter anxiety-related behavior as seen by no change in the number of entries in the open-arm of the EPM **(D)**, number of marbles buried **(E)** and number of entries in the center in the open field **(F)** compared to water controls. Long-term sucrose consumption does not induce depressive-like symptoms as seen by no differences in the immobility time in the forced swimming test **(G)** compared to water controls. Data are presented as mean ± SEM; *n* = 8 mice/group.

Following 5 weeks of sucrose consumption, one set of animals undertook five behavioral tests were conducted over 6 weeks (*n* = 8 per group; Figure 1A). These behavioral tests were conducted during dark cycle and were withdrawn from sucrose for 24 h. Elevated-plus-maze (EPM), a behavioral test used to observe anxiety-related behavior, was performed in an apparatus comprising of four arms, 2 open arms and 2 closed arms (35 cm × 5 cm), elevated 50 cm above the floor. The closed arms were fenced with 40 cm high walls. The experiment went for 5 min, with initial mouse placement in the center, facing the open arm. The number of entries and time spent in each arm was recorded using ANY-maze tracking software (Stoelting, IL, United States) (Walf and Frye, 2007; Belmer et al., 2018).

Novelty suppressed feeding (NSF) test measures the novelty induced anxiety/impulse control by assessing the latency to approach and eat familiar food in a novel/aversive environment. Animals were food-deprived for 16 h before being placed in an open area (L = 40 cm, W = 36 cm, H = 18 cm) with new bedding and a piece of familiar chow in the middle of the area. Latency to feed was measured in seconds before the animal eats the food, by two experimenters blind to the diet (Bevilacqua et al., 2010).

Marble burying (MB) is used to test anxiety and obsessive-compulsive disorder-like behavior. MB was performed in novel individual plastic cages (21 cm × 38 cm × 14 cm) containing 5 cm thick sawdust bedding. Ten glass marbles (diameter 10–12 mm) were evenly spaced in 2 rows of 5 marbles on the bedding. After 20 min, the number of unburied marbles was averaged from counting by two experimenters. A marble covered at least two-third (2/3) of its size by saw dust will be considered as “buried”(Deacon, 2006; Belmer et al., 2018).

Open-field (OF) test is used to measure exploratory behavior, general locomotor behavior and anxiety. OF was performed in an open arena of 30 cm × 30 cm × 40 cm. The floor was divided into 16 equal squares (7 cm × 7 cm) and a central region of 10 cm × 10 cm was considered as the center, outside this central region was considered as the periphery. Mice were initially placed in one corner, and allowed to explore freely for 10 min. The number of entries and the time spent in the center or periphery were recorded using the ANY-maze software (Bailey and Crawley, 2009; Belmer et al., 2018). General locomotor activity was assessed using the open field apparatus and recorded using ANY-maze software.

The forced swimming test (FS) is commonly used to test the efficacy of antidepressants and by extension, to assess depressive-like behaviors. The FS test was conducted in a cylindrical glass container measuring 50 cm in height and a diameter of 20 cm. The immobility time was recorded using ANY-maze tracking software (Yankelevitch-Yahav et al., 2015).

### Memory Assessment

Using a separate group of animals on the same drinking protocol, two recognition/spatial memory tests were conducted (*n* = 15 per group, Figure 3A). After 8 weeks of 25% sucrose consumption, recognition (episodic) memory was assessed using novel object recognition (NOR) test. NOR protocol ran over 4 days and was performed in the open field apparatus. On the first day, mice were habituated to the open field apparatus for 10 min. The second and third day, mice were presented with two identical objects for 10 min. No animals were excluded from analysis based on their object exploration times. On the last day, under 24 h withdrawal to 25% sucrose solution, one of the two familiar objects was replaced with a novel object (Figure 3D) and the interactions with the objects, including metrics such as object exploration time and latency to reach the novel and familiar objects were recorded on ANY-maze software (Leger et al., 2013).

The same animals continued to drink 25% sucrose for 2 more weeks (Week 10) before spatial recognition was measured using the Morris Water Maze. This test evaluates the animal’s ability to escape a stressful situation in a large pool of water. The heated pool (22°C; 150 cm diameter) was divided into 4 quadrants with a designated visual cue in each quadrant (Figure 3G). We used a 4-day protocol (Flores-Ramirez et al., 2019) starting with a pre-training/habituation day where the location of the escape platform was introduced with the platform being visible 1 cm above the water surface. Each animal was placed on the platform for 10 s before being released into the water facing the platform, less than 10 cm away. Once the platform was reached, place mouse onto the platform for 10 s then released again at a greater distance (20 cm). This was repeated for a third time with the release greater again (30 cm). If platform was not reached, experimenter gently guided the mouse to the platform and the animal was released in the water again until the mouse swam to the platform unaided. On day 2 (training day), the platform was partially covered by water and odorless non-toxic paint was added so the platform was not visible. Each animal had 8 trials of 60 s duration to swim to the platform, being released from each quadrant while facing the wall twice in a random order. Between trials the mouse had a 45 s break before the next trial commenced in a warmed holding cage. On day 3 (testing day), performed under 24 h withdrawal from 25% sucrose solution, each animal was released from the furthest quadrant to reach the platform and were removed from the pool if they did not reach the platform in 60 s. The time spent to reach platform was recorded manually having up to 1 min to reach the platform. Testing videos was recorded using ANY-maze. Day 4 involved probing the anima’s memory, with the platform being removed and the amount of time spent in the quadrant or in platform area recorded using ANY-maze. Heating pads and lamps were used and readily available across all four days.

### Neurogenesis

Following 12 weeks of sucrose drinking, a total of three intraperitoneal injections of the cell proliferation marker, 5-ethynyl-2′-deoxyuridine (EdU; 50 mg/kg) were administered over 2 weeks (days 0, 7, and 15) as previously described (Belmer et al., 2018; Patkar et al., 2019). This dose has been reported to label all actively dividing precursors in the mouse subgranular zone (Mandyam et al., 2007). One week after the last EdU injection, animals were deeply anesthetized with sodium pentobarbital (100 mg/kg, Lethobarb, Virbac, Australia) and transcardially perfused with 4% paraformaldehyde. Brains were harvested and postfixed in the same fixative overnight at 4°C. Thirty micron-thick coronal vibratome sections were collected and kept floating in ice-cold 0.1M phosphate buffer saline (PBS). Sections containing the hippocampus were selected for immunohistochemistry. The sections were permeabilized in 1% Triton X100, 0.1% Tween20 in PBS for 1 h at room temperature and incubated with EdU Click-iT^TM^ EdU Alexa Fluor^TM^ 488 Imaging Kit as per supplier recommendation (Thermo-Fisher Scientific, C10637). When required, antigen retrieval (0.05% Tween-20 in 10 mM sodium citrate, pH 6, 5–15 min at 80°C) was performed. After three washes in PBS, sections were incubated in blocking solution (2% normal goat serum, 0.3% Triton X100 and 0.05% Tween-20) for 1 h at room temperature. The sections were then probed for markers of each stage of neurogenesis (Figure 3A) and incubated with primary antibodies, diluted in blocking solution, for 24 h at room temperature, washed 3 times in blocking solution and then incubated with corresponding secondary antibodies, for 2 h at room temperature (Supplementary Table 1). When biotinylated secondaries were used, sections were incubated in streptavidin-CY3 for 30 min at room temperature. Sections were mounted in Prolong gold antifade mountant with DAPI (Thermofisher Scientific).

### Imaging and Analysis

Four coronal sections of whole dentate gyri per animals were imaged on the Leica DMi8 SP8 Laser Point Scanning confocal microscope using a 40 × objective (NA 0.85), x0.5 numerical zoom and 0.5 z-step. Consecutive sections were used across all staining groups. Images were deconvolved using Huygens professional v16.10 (Scientific Volume Imaging, Netherlands) and converted in.tif for subsequent quantification in Neurolucida 360 (MBF Bioscience). Early stages of neurogenesis were counted: stage 1: EdU^+^/GFAP^+^/Nestin^–^; stage 2: EdU^+^/Nestin^+^/GFAP^–^ and stage 3: EdU^+^/DCX^+^ as well as glial cell types: astrocytes (EdU^+^/GFAP^+^), microglia (EdU^+^/IBA-1^+^) and oligodendrocytes (EdU^+^/Olig 2^+^), see Figure 4A. Quantification was performed by an experimenter blind to the treatment, averaged per animal and plot as mean ± SEM for each group. Density of counted cells was normalized to the volume of granular cell layer sampled in each group as previous described in Belmer et al. (2018) and Patkar et al. (2019).

### Statistics

Comparisons between groups were statistically analyzed using *t*-test, one-way or two-way ANOVA, as appropriate, followed by a Bonferroni-multiple comparison *post hoc* test using GraphPad Prism 8 (Graph Pad Software Co., CA, United States). *P* values <0.05 were considered significant. All values are expressed as the mean ± SEM.

## Results

### Long-Term Sucrose Intake Increases Weight Gain

We assessed the effects of long-term unrestricted access to sucrose on body weight over 12 weeks (60 exposures days). After 12 weeks of access to 25% sucrose, mice exhibited stable levels of sucrose intake around 80–90 g/kg/day (mean 82.2 ± 0.9 g/kg/24 h indicated by the red line; Figure 1B). A significant increase in overall weight was observed, starting around 4 weeks and increasing throughout the 12 weeks of exposure (Figure 1C, Mixed effect repeated measure two-way ANOVA, F_(__58_, _1276__)_ = 9.265, *p* = 0.0001) until reaching 10.6% overweight (Bonferroni multiple comparison: 33.22 ± 0.85 g vs 30.03 ± 0.59 g, *p* < 0.0002) compared to water controls.

### Long-Term Sucrose Intake Does Not Produce Anxiety- and Depressive-Like Behavior

Sugar-withdrawn rats consistently exhibit both anxious and depressive-like symptoms (Vollmayr et al., 2004; Avena et al., 2008; Iemolo et al., 2012; Parylak et al., 2012; Harrell et al., 2015; Eudave et al., 2018; Santos et al., 2018). One study in C57BL/6 J mice consuming high levels of 10% sucrose (around 72 g/kg/24 h) for 4 weeks, found increased anxiety- (EPM) and depressive-like behavior (tail suspension test) after one week of withdrawal (Kim et al., 2018). To evaluate the effect of higher level of sugar intake (around 85 g/kg/24 h) after 6 weeks of exposure on emotional behavior, we assessed withdrawal-induced anxious and depressive-like behaviors in the EPM, MB, OF, and FS tests, 24 h after the last drinking session of the week. The number of open arm entries within 5 min was similar in the EPM, between sucrose and water control animals suggesting no change in anxiety-related behavior (Figure 1D, ns, *p* = 0.5690, *t*-test). The MB test also yielded a similar result with water- and sugar-exposed animals burying a similar amount of marbles (Figure 1E, ns, *p* = 0.9062, *t*-test). The OF test showed no difference in the number of entries into the center between sucrose and water exposed animals, hence confirming the absence of anxiety-like behavior following long-term unrestricted access to sucrose (Figure 1F, ns, *p* = 0.3583, *t*-test). No change in immobility time in the FS was evident in sucrose consuming animals suggesting no depressive-like symptoms (Figure 1G, ns, *p* = 0.6654, *t*-test).

### Long-Term Sucrose Intake Produces Hyperactivity/Hyperlocomotion

There is growing evidence showing that addiction and substance dependence strongly rely on increased arousal, hyperactivity, impulsivity and compulsion following cessation (Crews and Boettiger, 2009). To understand how sucrose overconsumption affects hyperactivity and compulsion, we assessed general locomotor activity using OF. To evaluate impulse control we used the NSF, conflict based anxiety test that has previously been shown to reflect impulsivity-like behavior (Bevilacqua et al., 2010; Angoa-Pérez et al., 2014; Piggott et al., 2020).

**FIGURE 2:**
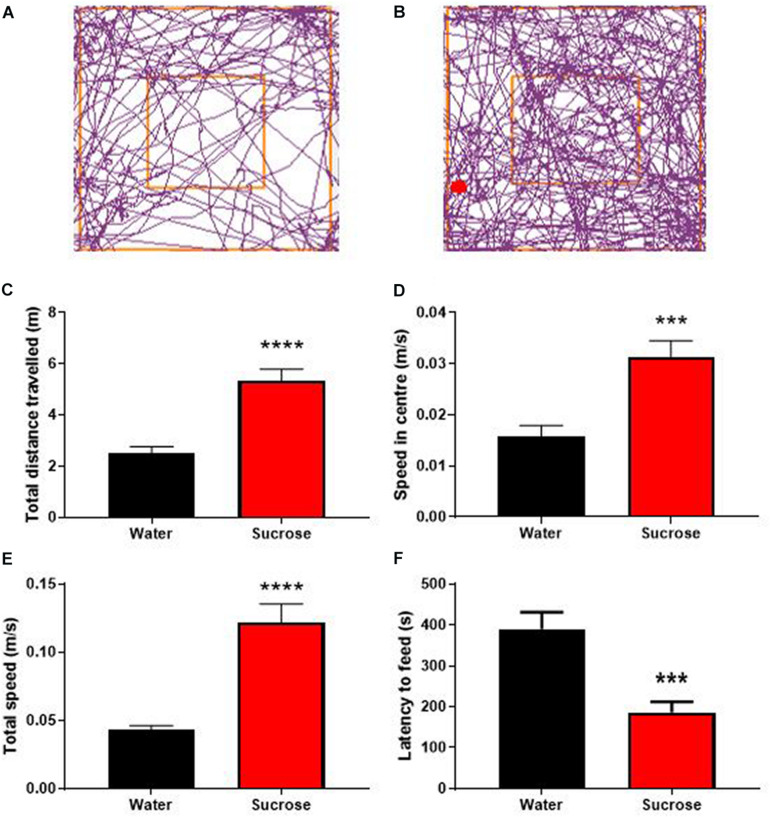
Long-term sucrose consumption produces hyperactivity and reduces the control to resist food. Tracking plot of water- **(A)** and sucrose-consuming mice **(B)** illustrating that long-term sucrose consumption increases the total distances traveled in the open field **(C)**, the speed in the center **(D)** and total speed **(E)** in the open-field compared to water controls. Long-term sucrose consumption also reduced the latency to feed suggesting reduced control to resist food **(F)**. Data are presented as mean ± SEM; *n* = 8 mice/group. ***p < 0.001, ****p < 0.0001.

Sucrose consuming mice displayed a higher general locomotor activity as observed by an increase in total distance traveled (Figures 2B,C) compared to water control animals (Figures 2A,C, *p* < 0.0001, *t*-test). This hyperlocomotion was accompanied by hyperactivity as evidenced by increased average speed in center of the open-field apparatus (Figure 2D, *p* = 0.0008, *t*-test), total average speed (Figure 2E, *p* < 0.0001, *t*-test) in the OF apparatus, and reduced latency to feed in the NSF, compared to water controls (Figure 2F, *p* = 0.0005, *t*-test). Combined with hyperactivity, this lack of novelty-induced feeding inhibition could be interpreted as a reduction of impulse control to anxiogenic environment and further suggests that chronic sucrose consumption produces both hyperactivity and impulsivity (Bevilacqua et al., 2010; Angoa-Pérez et al., 2014; Piggott et al., 2020).

**FIGURE 3:**
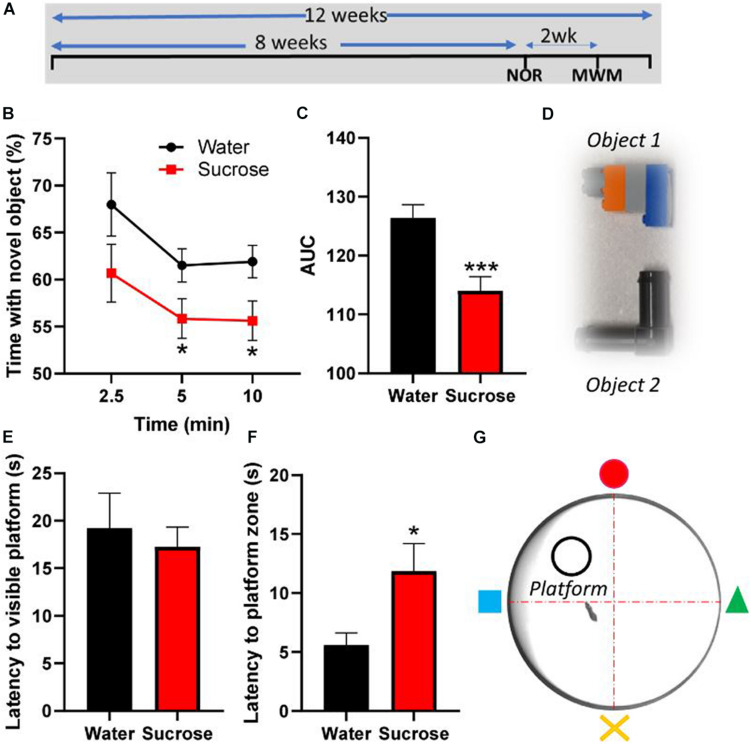
Long-term sucrose consumption alters episodic and spatial memory. **(A)** Experimental design of the testing of episodic and spatial memory in the novel-object recognition (NOR) and Morris-watermaze (MWM) tests. Animals consumed 25% sucrose for 8 weeks prior to behavioral testing and continued to be exposed to sucrose for a total of 12 weeks. Memory tests were conducted 3 weeks apart. Animals were assigned into two groups: sugar-withdrawn animals and water controls. **(B–D)** Long-term sucrose drinking animals interact less with a novel object across the duration of the test, compared to water controls **(B)**, as confirmed by a reduction in the area-under-curve (AUC) **(C)**. The 2 objects used alternatively in the novel-object-recognition test, which are discernible in shape, size and color, are pictured in panel **(D)**. **(E–G)** Long-term sucrose consuming mice showed no alteration in their learning and swimming behavior to reach a visible platform **(E)**, however, they showed an increased latency to reach the zone where the platform was previously placed **(F)** compared to water controls. Schematic drawing of the MWM apparatus used is depicted in panel **(G)**. Data are presented as mean ± SEM; *n* = 8 mice/group. *p < 0.05, ***p < 0.001.

**FIGURE 4:**
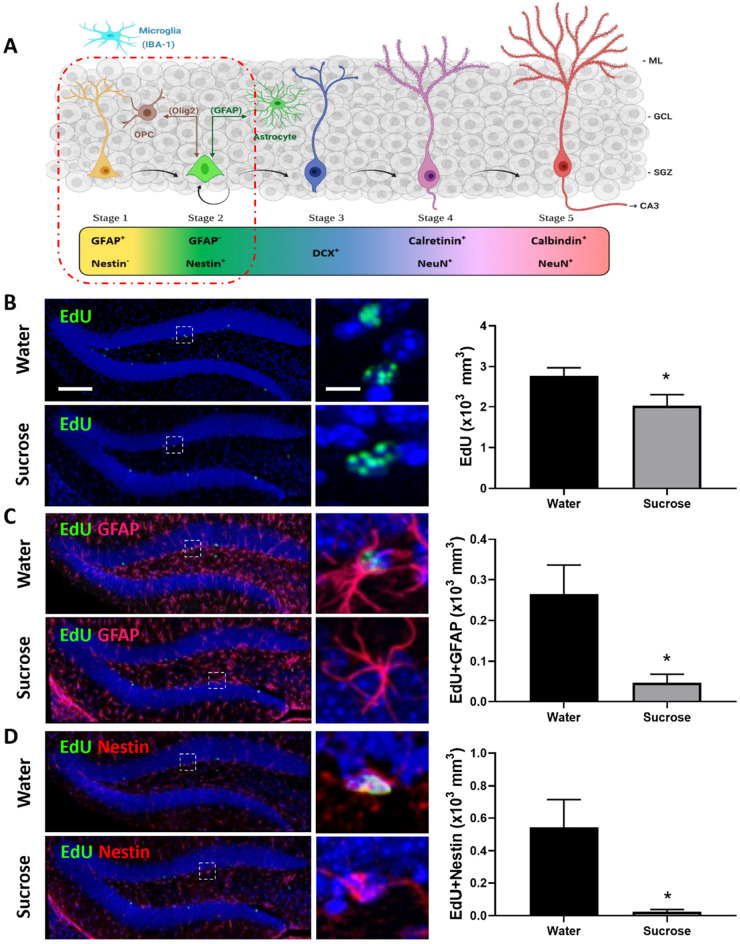
Long-term sucrose consumption reduces the early phases of hippocampal neurogenesis. **(A)** Stages of neurogenesis in the dentate gyrus of the hippocampus. In stage 1 (proliferation phase; in yellow), the newly generated cells express glial fibrillary acidic protein (GFAP) and are putative progenitor/stem cells located in the subgranular zone (SGZ). The cells in stage 2 (differentiation phase; in green) will lose their GFAP and start expressing Nestin. This determines their fate in the neuronal lineage. Stage 1 progenitors not only give rise to newborn neurons, they can also turn into glial cells. Glial cells [astrocyte and oligodendrocyte progenitor cell (OPC)] can convert back into newborn neurons here in stage 2 and vice-versa. In stage 3, the immature neurons express doublecortin (DCX; in cobalt blue) and have started to migrate into the granule cell layer (GCL) of the dentate gyrus. As the neuron matures, it will send its dendrites toward the molecular layer (ML) of the dentate gyrus and extend their axonal projections toward the hippocampal CA3 pyramidal cell layer and will start losing their DCX and start expressing postmitotic neuronal marker NeuN and calretinin (stage 4; magenta). As the neuron establishes synaptic contacts from the entorhinal cortex and it sends output to the CA3 and hilus regions of the hippocampus the neuron is classified as in stage 5 (in cherry red). Stage 5 neurons start expressing calbindin and continue expressing NeuN. Unlike the astrocytes and oligodendrocytes that are derived from the neuroectoderm, microglia (in cyan blue) are neuroglia derived by embryonic mesoderm. Abbreviations used: GFAP, glial fibrillary acidic protein; OPC, oligodendrocyte progenitor cell; Olig2, oligodendrocyte lineage transcription factor 2; IBA-1, ionized calcium binding adaptor molecule 1; DCX, doublecortin; NeuN, Fox-3, Rbfox3, or Hexaribonucleotide Binding Protein-3; SGZ, subgranular zone; GCL, granule cell layer; ML, molecular layer [original drawing, created using Biorender, adapted from Lucassen et al. (2010)]. **(B)** Long-term sugar consumption reduced the density of EdU positive cells (green) in the dentate gyrus of the hippocampus. **(C)** There was a reduction in the density of EdU^+^ (green)/GFAP^+^ (red) immunoreactive cells indicating a reduction in stage 1 (putative stem cells) neurogenesis. **(D)** A reduction was also observed in the number of EdU^+^ (green)/Nestin^+^ (red)-immunoreactive cells indicating a reduction in stage 2 (neuronal progenitors). All images are colocalized with DAPI (blue). Data are presented as mean ± SEM; *n* = 8 mice/group. *p < 0.05. Representative image scale bar is 100 μm and close up representative image scale bar is 10 μm.

### Long-Term Sucrose Intake Alters Both Episodic and Spatial Memory

Since hyperactivity correlates with memory impairments (Ortega et al., 2020), we assessed the consequences of chronic sucrose consumption and subsequent hyperactivity on learning and two types of memory, episodic and spatial, using the NOR and MWM tests, respectively. Although sucrose consuming mice learn to discriminate between old and new objects, the proportion of interaction time with the novel object compared to old object was significantly lower than water control mice at 5 min (*p* = 0.048) and 10 min (*p* = 0.028) following the presentation of the objects (Figure 3B, Two-way ANOVA, F _(__1_, _84__)_ = 10.35, *p* = 0.0018), hence showing a significant reduction of the area under curve (Figure 3C, *t*-test, *p* = 0.0008).

In the MWM, sucrose and water drinking mice showed similar latency to reach a visible platform, confirming that, albeit sucrose mice being slightly overweight compared to water controls, there was no alteration in learning or their swimming behavior (Figure 3E, *t*-test, *p* = 0.63). However, when the platform was hidden, sucrose-consuming mice took longer than water consuming mice to reach the platform area (Figure 3F, *t*-test, *p* = 0.025). These results suggest that chronic overconsumption of sucrose alters both episodic and spatial memory without affecting the learning process.

### Long-Term Sucrose Intake Decreases Hippocampal Cell Proliferation and Neurogenesis

Anxiety, depression, and cognitive deficits are strongly associated with alteration in hippocampal neurogenesis. Although, sucrose did not elicit anxiety- or depression-like behavior, we assessed whether sucrose-induced deficits in memorization was associated with changes in hippocampal neurogenesis (Figure 4A). Mice chronically consuming a highsucrose diet showed a reduction in the overall density of dentate gyrus proliferating cells (EdU^+^) compared to water controls (Figure 4B, *p* = 0.0332, *t*-test). This decrease in cell proliferation was likely mediated by decreased neurogenesis, as evidenced by a reduction in the density of both putative stem cells (stage 1: EdU^+^/GFAP^+^/Nestin^–^; Figure 4C, *p* = 0.0127, *t*-test) and neuronal progenitors (newborn neurons, stage 2: EdU^+^/GFAP^–^/Nestin^+^; Figure 4D, *p* = 0.0106, *t*-test) compared to water controls, suggesting that continuous sucrose intake alters the transition or differentiation of progenitors into the proliferating phase. No change within differentiated neuroblasts was observed (stage 3; EdU^+^/DCX^+^; *p* = 0.2309, *t*-test, not shown) suggesting sucrose consumption principally affects the earlier stages of neurogenesis.

### Restricting the Availability of Sucrose Dampens the Neurocognitive Deficits

**FIGURE 5:**
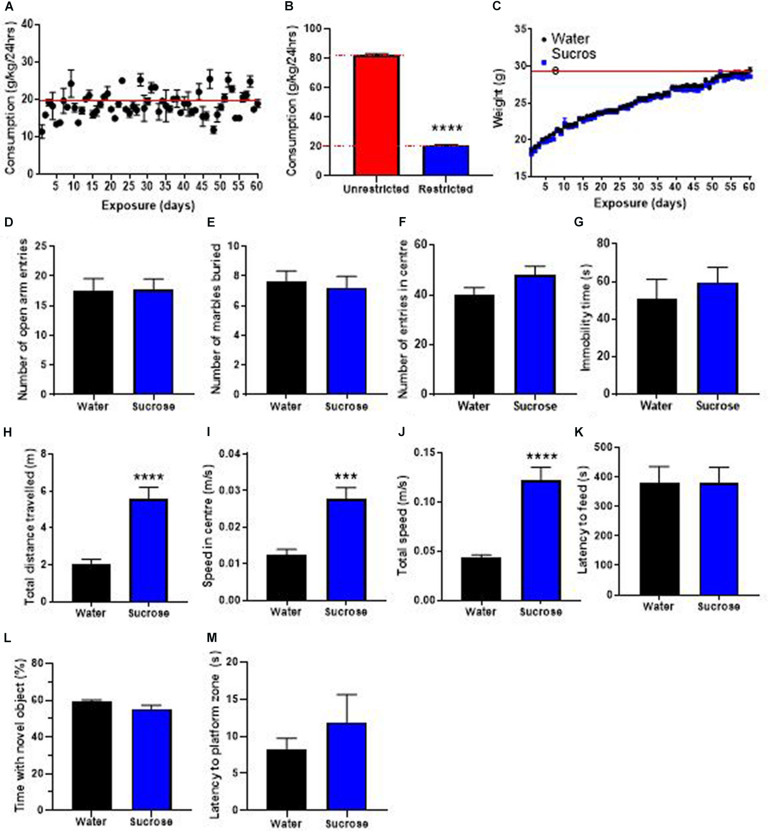
Restricting access to sucrose consumption results in an absence of increased weight gain with no emotional and cognitive alterations. **(A)** Mice exhibit stable levels of long-term sucrose intake of 20.9 ± 0.3 g/kg indicated by the red line. This level of sucrose intake is four-fold lower than when sucrose access was unrestricted **(B)** and was not associated with any changes in weight gain **(C)**, or anxiety-related behavior as seen by no change in the number of entries in the open-arm of the EPM **(D)**, the number of marbles buried **(E)** and the number of entries in the center in the open field **(F)** compared to water controls. Restricting long-term sucrose consumption did not induce depressive-like symptoms as seen by no differences in the immobility time in the forced swimming test **(G)** compared to water controls. Restricted sucrose consumption increases the total distances traveled in the open field **(H)**, the speed in the center **(I)** and total speed **(J)** in the open-field, however, there was no change in the latency to feed **(K)**, the time spent with a novel object **(L)** and the latency to reach the zone where the platform was previously placed **(M)**, compared to water controls. Data are presented as mean ± SEM; *n* = 8 mice/group. ***p < 0.001, ****p < 0.0001.

The WHO’s guidelines recommend restricting the availability of sugar in the current diet and advocate a four-time reduction of the daily intake of sugar. Therefore, we investigated the consequences of restricting access to sucrose solution to only 2 h/day, on weight gain, locomotion, emotion, cognition, and neurogenesis. Mice showed a daily intake of sucrose of 20.9 ± 0.3 g/kg (Figure 5A), about 4 times less than when access was unrestricted (Figure 5B, *t*-test, *p* < 0.0001). This lower daily intake was associated with no change in weight gain over 12 weeks of exposure, compared to water controls (Figure 5C, Two-way ANOVA, F _(__1_, 11) = 0.3511, *p* = 0.5655).

We then evaluated if restricting access to sucrose alters anxiety- and depression-related behavior in the same behavioral tests. Restricting the access to sucrose to 2 h per day did not elicit any change in anxiety-like behavior, as evidenced by no difference in the number of open-arm entries in the EPM (Figure 5D, *t*-test, *p* = 0.95), no difference in the number of marbles buried (Figure 5E, *t*-test, *p* = 0.69) and no difference in the number of entries in the center of the OF (Figure 5F, *t*-test, *p* = 0.11). No difference was observed in the immobility time in the FS (Figure 5G, *t*-test, *p* = 0.51) suggesting that restricting access to sucrose does not elicit depressive-like behavior.

Interestingly, restricting access to sucrose still elicited hyperactivity as shown by significant increases in the total distance traveled (Figure 5H, *t*-test, *p* < 0.0001), the speed in the center (Figure 5I, *t*-test, *p* = 0.0002) and total speed (Figure 5J, *t*-test, *p* < 0.0001) in the OF, compared to water control animals. However, there was no change in the latency to feed compared to water controls (Figure 5K, *t*-test, *p* = 0.37). These results suggest that restricting sucrose availability and reducing overall daily intake does not affect the inhibitory control to resist food, despite promoting hyperactivity. This increased hyperactivity with no deficits in control to resist food was not accompanied by any alteration in memory, as evidenced by no change in the proportion of time interacting with a novel object (Figure 5L, *t*-test, *p* = 0.97) or the latency to reach the platform area in the MWM (Figure 5M, *t*-test, *p* = 0.10). This absence of memory deficits was accompanied by no change in hippocampal neurogenesis, however, an increased oligodendrogenesis was observed in the dentate gyrus (Supplementary Figure 1).

## Discussion

Sugar became embedded in the food and beverage chains, leading to overconsumption in children and adolescents (Han and Powell, 2013; Dereñ et al., 2019). It is therefore important to investigate how long-term intake of sugar, starting at adolescence, leads to long-term effects into adulthood. The present study shows there are deleterious effects of long-term sugar intake, on weight gain, hyperactivity, impulsivity, and deficits in memory and hippocampal neurogenesis.

While the WHO recommends that the amount of sugar in sugar-sweetened be reduced by four-fold to decrease the risk of childhood overweight and obesity (WHO, 2019), the impact of sugar intake on the rise in obesity rates is still debated (Alexander Bentley et al., 2020). Indeed, overall sugar consumption has dropped since the mid-1990’s whereas the obesity rate has continuously increased. It has been proposed that this rise in obesity could result from a delayed effect of excess sugar, suggesting that adult obesity could be driven by high sugar intake over a life span (Alexander Bentley et al., 2020). There are many ways that a decrease in overall sugar consumption and an increase in obesity rates can be viewed. Sugar has hidden properties, activates the hypothalamus, inhibits ghrelin and leptin, leading to over-eating other types of foods (Jacques et al., 2019). It has been shown that both obesity and sweet-taste can be passed on through epigenetic modification (Öst et al., 2014; Donkin et al., 2016; Ling and Rönn, 2019). This means that once obesity or sugar preference has been established, it can be passed on in families for up to 3 generations going forward.

In line with this, we observed that unrestricted access to sugar, beginning at adolescence, only starts to affect weight gain into adulthood, after 4–5 weeks of sucrose consumption. Although rodent models of diet-induced obesity use body weight as a measure of obesity, the effects of high-sugar diets on body weight are inconsistent (Jurdak et al., 2008; Jurdak and Kanarek, 2009). While we observed a significant increase in weight gain in sucrose consuming animals, we cannot comment on the potential link between obesity and long-term unrestricted intake of sugar. Further investigation of metabolic marker expression, such as adiposity, insulin resistance, leptin, adiponectin, will be needed to elucidate how long-term sucrose consumption predisposes to obesity. Interestingly, we found that a 4-times reduction of daily sucrose intake is able to prevent sugar-induced increase in weight gain, supporting the WHO’s guideline to reduce the impact of sugar on the rise of obesity rate.

We showed that long-term sucrose consumption did not produce any anxiety- or depression-related behavior, although previous studies have shown that acute and chronic withdrawal from sucrose can induce anxious and depressive like behaviors (Colantuoni et al., 2002; Avena et al., 2008; Iemolo et al., 2012; Parylak et al., 2012; Harrell et al., 2015; Eudave et al., 2018; Kim et al., 2018; Santos et al., 2018). Anxiety- and depression-like behaviors are likely present if withdrawal follows extensive periods of sucrose consumption. Short-term exposure to sucrose (<1 month) does not lead to increased anxiety-like behavior in rats (Parylak et al., 2012) while longer exposure (>1 month) results in increased anxiety-like behavior 24 h after withdrawal (Colantuoni et al., 2002; Avena et al., 2008). Interestingly, short-term unrestricted access to 10% sucrose induced depression- and anxiety-like behavior after one week withdrawal (Kim et al., 2018). Since the aforementioned studies used different models of sucrose consumption with concentrations of sucrose solutions ranging from 7.9 to 35%, we cannot rule out an absence of anxiety-like behavior in our study due to methodological or interspecies differences. In addition, many studies incited a binge-like patterns of sugar (10% sucrose or 25% glucose) consumption by food-depriving the animals for 12 h before the sucrose drinking sessions. The food deprivation/restriction introduced in these studies may have changed motivational states and reward seeking behavior, adding another level of psychological stress (Toth and Gardiner, 2000). Strikingly, longer sucrose exposure (>3 months) showed no significant effects on anxiety- and depression-like behavior in rats (Cao et al., 2007; Chepulis et al., 2009), hence suggesting that long term (i.e., greater than 1 month but less than 3 months) consumption of sucrose increases anxiety-like behavior, followed by a return to baseline levels of anxiety- and depression-related behavior at 3 months.

Overall, we observed no anxious or depressive behavior in mice after 6–10 weeks of sucrose consumption. The reliability of our findings is bolstered by the fact that we used two different models, restricted and unrestricted. Perhaps sugar does not produce the same emotional deficits between species. An explanation could be due to differences metabolism, with a well-described faster metabolism in mice compared to rats (Radermacher and Haouzi, 2013). It may also be possible that higher concentrations of sucrose (greater than 10%) recruits different/additional neural circuits, involving other behavioral deficits, such as hyperactivity and/or impulsivity that mask the behavioral inhibition produced by anxiogenics cues/environments.

Our results showed that long-term sucrose overconsumption increases basal locomotor activity, which could be interpreted as hyperactivity. There are limited studies examining the effect of sucrose on locomotor activity, with, to the best of our knowledge, only one study reporting no change in locomotor activity in rats (Avena and Hoebel, 2003a). Our observation of sugar-induced hyperactivity prompted the investigation of the effect of long-term sugar consumption on impulse control and inhibitory control to resist food. Interestingly, long-term sucrose consumption reduced impulse control in the novelty-suppressed feeding, and this was not observed when sucrose access was restricted. Although novelty-suppressed feeding test is primarily to assess anxiety-like behaviors, studies have shown that a reduced latency to feed could be correlated with augmented food seeking and increased meal size (Biddinger et al., 2020), increased hunger and reduced-feeding control after fasting (Burghardt et al., 2016), and together with hyperlocomotion, increased motor impulsivity (Bevilacqua et al., 2010; Angoa-Pérez et al., 2014; Piggott et al., 2020). This suggests that the reduced latency to feed we observed after long-term sucrose intake could be the result of impulsivity. However, there remains disparity in the literature regarding sugar’s effect on impulsivity in rats with studies suggesting sugar does not (Stein et al., 2015; Wong et al., 2017) and others supporting sugar does produce impulsivity (Steele et al., 2017). Therefore, further investigation is required to identify the mechanism underlying the effect of sugar on locomotor and impulsive behavior in mice. This could be explored further using delay discounting test (temporal discounting) or 5-choice serial reaction time task (visual attentional processes and impulse control) (Reichelt et al., 2015, 2016; Lemos et al., 2016).

Excessive sucrose consumption in adolescent rats has been associated with deficits in spatial memory or object recognition memory (Reichelt et al., 2015, 2016; Lemos et al., 2016), and this could be principally mediated by the fructose component of sucrose (Hsu et al., 2015). However, the link between memory deficits and changes in hippocampal neurogenesis following long-term sucrose consumption has been relatively unexplored. In our study, reduction in hippocampal neurogenesis was only observed when memory deficits were observed, for example, when access to sucrose was unrestricted.

Indeed, no change in hippocampal neurogenesis was observed when sucrose access was restricted, and memory not affected. Our results further suggest that unrestricted sucrose consumption likely affects neurogenesis by reducing cell proliferation, generation of putative stem cell and survival/maturation of newborn neurons.

Reduced cell proliferation followed by reduced production of neuronal progenitors suggest reduced neurogenesis/turn over (Cisternas et al., 2015). This result is in accordance with previous ethanol studies in rodents suggesting sugar consumption is also intervening at the G1 phase of the cell cycle, changing the number of cells entering the S phase (Belmer et al., 2018; Patkar et al., 2019). Interestingly, we did not see an effect on neuroblast differentiation, suggesting the reduction in the initial number of putative stem cells able to dedifferentiate and proliferate. This could be due to the generation of pluripotent transit-amplifying progenitor cells [TAPs (Potten and Loeffler, 1990)] that remain in quiescence for long periods before differentiation (Doetsch et al., 2002). It is possible that sugar reduces the number of TAPs resulting in the changes observed here. Early-stage neurogenic deficits have not been observed previously. Our results found a reduction in putative stem cell and newborn neurons with no change in differentiated neuroblasts. This absence of effect of sugar on neuroblast differentiation has also been reported in rats with unrestricted access to sucrose, therefore confirming a degree of interspecies similarity. Another explanation of the reduction in proliferating cells is cell death. Unrestricted consumption of sugar has been shown to increase apoptosis (TUNEL) suggesting our reduction in proliferating cells could be due to neuronal death as a result of sugar overconsumption (van der Borght et al., 2011). Neuronal maturation and survival have been reported to be reduced in the dentate gyrus of the hippocampus after sugar consumption (van der Borght et al., 2011; Cisternas et al., 2015). However, it is possible their protocol of cell proliferation labeling (BrdU injection two weeks prior) has misevaluated neuronal maturation and survival as the maturation process of neurons can take up 1–2 months in rodents (Zhao et al., 2008).

A limitation of this investigation is that the results may not be applicable to female models of sugar overconsumption, as only male mice were tested. C57/Bl6 male mice have both increased overall activity and heightened anxiety when compared to female mice particularly in maternal separation and chronic stress models (Romeo et al., 2003; Veenema et al., 2007; Niwa et al., 2011), meaning that subtle differences in the behavior of male mice are more easily detected compared to female mice. Female sex hormones also influence appetitive signaling in the brain (Gao et al., 2007; Santiago et al., 2016), which therefore increases the methodological complexity of including female mice in this study, as all data would need to be normalized to the estrous cycle. Females are widely underrepresented in preclinical models of addiction (Beery and Zucker, 2011; Shansky, 2019), with a majority of studies in this field conducted exclusively on male mice. This is a major ongoing issue with neuroscience research, and future investigations should explore if similar behavioral consequences of sugar consumption are present in female rodents. However, we believe the results of this study provide foundational knowledge that can be extended upon to benefit addiction and obesity generally. Together, our study demonstrates that excessive sugar consumption starting at adolescence elicits profound locomotor and memory deficits in adulthood, that may mimic the hyperactivity and cognitive dysfunctions observed in attention deficits and hyperactivity-like disorders. More interestingly, our results reveal that restricting sugar consumption intake, as recommended by the WHO, might be effective in limiting the negative consequences of sugar on obesity, and locomotor and cognitive impairments.

## Data Availability Statement

The raw data supporting the conclusions of this article will be made available by the authors, without undue reservation.

## Ethics Statement

The animal study was reviewed and approved by The University of Queensland and The Queensland University of Technology Animal Ethics Committees under approval QUT/053/18.

## Author Contributions

KB, AB, and SEB were responsible for the study concept and design. KB and IAC carried out the drinking experiments. KB, AB, and IAC performed behavioral animal experiments, analyzed the data and interpreted the findings. KB designed and performed the immunohistochemistry experiments and acquired the images with the technical advice of SBW at SBMS facility at The University of Queensland. KB and AB drafted the manuscript and drafted the figures. KB and JW drafted the drawing in Figure 3. SEB, IAC, FC, JW, and AB reviewed and edited the manuscript. All authors have critically reviewed the content and approved final version for submission.

## Conflict of Interest

The authors declare that the research was conducted in the absence of any commercial or financial relationships that could be construed as a potential conflict of interest.

## Supplementary Material

Supplementary Figure 1 Restricting access to sucrose consumption augments hippocampal oligodendrogenesis. **(A,B)** Restricted sugar consumption increased the density of EdU positive cells (green) in the dentate gyrus of the hippocampus. **(C,D)** There was no change in the density of stage 1 putative stem cells [EdU^+^/GFAP^+^, **(C)**] or stage 3 immature neurons [EdU^+^/DCX^+^, **(D)**] suggesting no change in neurogenesis, however, it appears that the increased density of EdU^+^ proliferative cells was mediated by an increased density of oligodendrocytes progenitors [EdU^+^ (green)/Olig^+^ (magenta), **(E,F)**]. **(G)** There was no difference in the density of proliferative microglia in the dentate gyrus, following restricted sugar consumption, compared to water controls. All images are colocalized with DAPI (blue). Representative image scale bar is 100 μm and close up representative image scale bar is 10 μm.
