# Correction: Long-term overconsumption of sugar starting at adolescence produces persistent hyperactivity and neurocognitive deficits in adulthood

**DOI:** 10.3389/fnins.2025.1643834

**Published:** 2025-07-01

**Authors:** Kate Beecher, Ignatius Alvarez Cooper, Joshua Wang, Shaun B. Walters, Fatemeh Chehrehasa, Selena E. Bartlett, Arnauld Belmer

**Affiliations:** ^1^Addiction Neuroscience and Obesity Laboratory, School of Clinical Sciences, Translational Research Institute, Faculty of Health, Queensland University of Technology, Brisbane, QLD, Australia; ^2^Addiction Neuroscience and Obesity Laboratory, School of Biomedical Sciences, Translational Research Institute, Faculty of Health, Queensland University of Technology, Brisbane, QLD, Australia; ^3^School of Biomedical Sciences, University of Queensland, Brisbane, QLD, Australia

**Keywords:** sucrose, hyperactivity, neurocognitive deficits, neurogenesis, adulthood

There was a mistake in Figure 4 as published. The representative image previously shown in Figure 4B, top left position (water) has been moved to the Figure 4B, bottom left (sugar). The top left panel Figure 4B, has been replaced with the correct representative image for the water condition. In Figures 4B, 4C, and 4D, the positions of the dashed squares indicating regions of interest have been adjusted for accuracy. The higher-magnification images on the right side of each panel have been rotated to better align with the updated dashed square positions. The corrected Figure 4 appears below.

**Figure 4:**
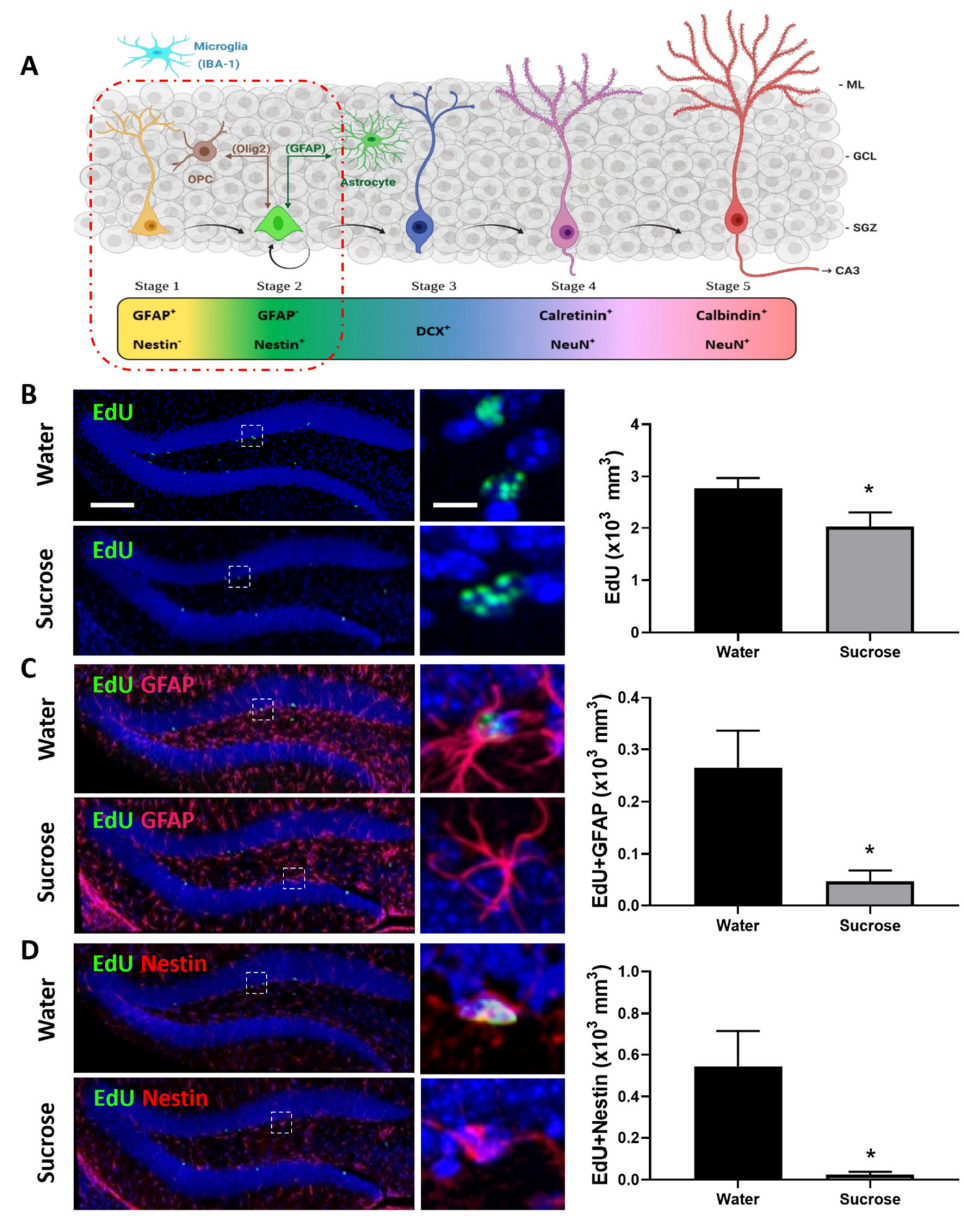
Long-term sucrose consumption reduces the early phases of hippocampal neurogenesis. **(A)** Stages of neurogenesis in the dentate gyrus of the hippocampus. In stage 1 (proliferation phase; in yellow), the newly generated cells express glial fibrillary acidic protein (GFAP) and are putative progenitor/stem cells located in the subgranular zone (SGZ). The cells in stage 2 (differentiation phase; in green) will lose their GFAP and start expressing Nestin. This determines their fate in the neuronal lineage. Stage 1 progenitors not only give rise to newborn neurons, they can also turn into glial cells. Glial cells [astrocyte and oligodendrocyte progenitor cell (OPC)] can convert back into newborn neurons here in stage 2 and vice-versa. In stage 3, the immature neurons express doublecortin (DCX; in cobalt blue) and have started to migrate into the granule cell layer (GCL) of the dentate gyrus. As the neuron matures, it will send its dendrites toward the molecular layer (ML) of the dentate gyrus and extend their axonal projections toward the hippocampal CA3 pyramidal cell layer and will start losing their DCX and start expressing postmitotic neuronal marker NeuN and calretinin (stage 4; magenta). As the neuron establishes synaptic contacts from the entorhinal cortex and it sends output to the CA3 and hilus regions of the hippocampus the neuron is classified as in stage 5 (in cherry red). Stage 5 neurons start expressing calbindin and continue expressing NeuN. Unlike the astrocytes and oligodendrocytes that are derived from the neuroectoderm, microglia (in cyan blue) are neuroglia derived by embryonic mesoderm. Abbreviations used: GFAP, glial fibrillary acidic protein; OPC, oligodendrocyte progenitor cell; Olig2, oligodendrocyte lineage transcription factor 2; IBA-1, ionized calcium binding adaptor molecule 1; DCX, doublecortin; NeuN, Fox-3, Rbfox3, or Hexaribonucleotide Binding Protein-3; SGZ, subgranular zone; GCL, granule cell layer; ML, molecular layer [original drawing, created using Biorender, adapted from Lucassen et al. (2010)]. **(B)** Long-term sugar consumption reduced the density of EdU positive cells (green) in the dentate gyrus of the hippocampus. **(C)** There was a reduction in the density of EdU^+^ (green)/GFAP^+^ (red) immunoreactive cells indicating a reduction in stage 1 (putative stem cells) neurogenesis. **(D)** A reduction was also observed in the number of EdU^+^ (green)/Nestin^+^ (red)-immunoreactive cells indicating a reduction in stage 2 (neuronal progenitors). All images are colocalized with DAPI (blue). Data are presented as mean ± SEM; *n* = 8 mice/group. *p < 0.05. Representative image scale bar is 100 μm and close up representative image scale bar is 10 μm.

The original article has been updated.

