# Supplementary material for: Long-Term Overconsumption of Sugar Starting at Adolescence Produces Persistent Hyperactivity and Neurocognitive Deficits in Adulthood
Source: Front Neurosci. 2021 Jun 7;15:670430. doi: 10.3389/fnins.2021.670430 (PMC8215656; doi:10.3389/fnins.2021.670430)
Supplement: Supplementary file 1 [file Data_Sheet_2.PDF]

Supplementary Table 1: Antibodies and concentrations.

<table>
<tr><th>Neurogenesis Stages</th><th>Cell Markers</th><th>Primary Antibody</th><th>Secondary Antibody</th></tr>
<tr><td>Stage 1: putative stem cells</td><td>EdU^{+} / GFAP^{+} /Nestin^{-}</td><td rowspan="2">-Rabbit anti-GFAP (Dako, ZO334, 1:500)
-Mouse anti-Nestin (Millipore, MAB353, 1:200)</td><td rowspan="2">-Donkey anti-Rabbit IgG, Alexa Fluor 647 (Invitrogen, A-31573, 1:500)
-Horse anti-Mouse IgG, Biotinylated (Vector labs, 1:200) + Streptavidin-CY3 (Invitrogen, #438315, 1:1000)</td></tr>
<tr><td>Stage 2: early differentiation</td><td>EdU^{+} / Nestin^{+} / GFAP^{-}</td></tr>
<tr><td>Stage 3: differentiated neuroblast</td><td>EdU^{+} / DCX^{+}</td><td>-Rabbit anti-DCX (Abcam #18723, 1:200)</td><td>-Goat anti-Rabbit IgG, Alexa Fluor 594 (Invitrogen, A-11012, 1:500)</td></tr>
<tr><td>Glia</td><td>IBA-1^{+} / Olig2^{+}</td><td>-Goat anti-Iba1 (Abcam, AB5076, 1:500)
-Rabbit anti-Olig 2 (Millipore, AB9610, 1:500)</td><td>-Donkey anti-Goat IgG, Alexa Fluor 594 (Invitrogen, A32758, 1:500)
-Donkey anti-Rabbit IgG, Alexa Fluor 647 (Invitrogen, A-31573, 1:500)</td></tr>
</table>
