# Supplementary figures and images for: Long-Term Overconsumption of Sugar Starting at Adolescence Produces Persistent Hyperactivity and Neurocognitive Deficits in Adulthood
Source: Front Neurosci. 2021 Jun 7;15:670430. doi: 10.3389/fnins.2021.670430 (PMC8215656; doi:10.3389/fnins.2021.670430)

**A**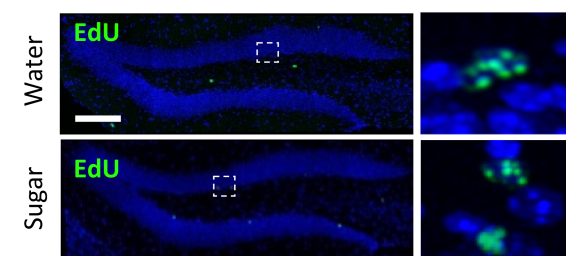**B**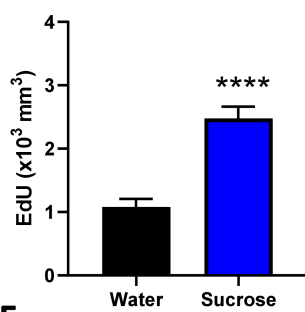**C**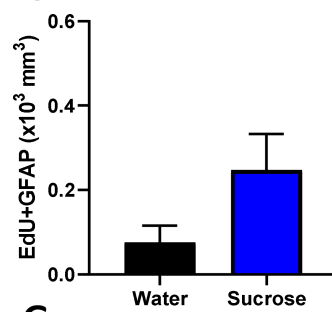**D**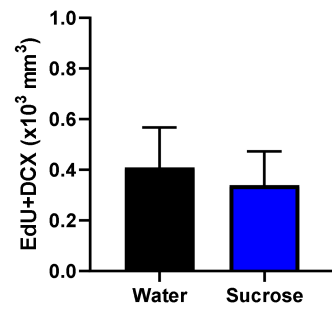**E**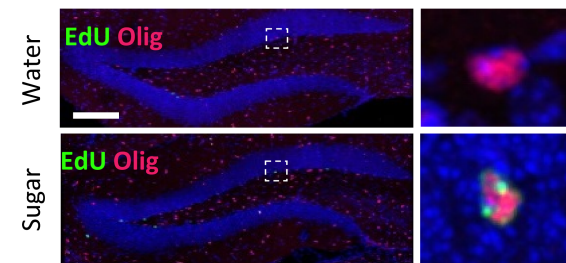**F**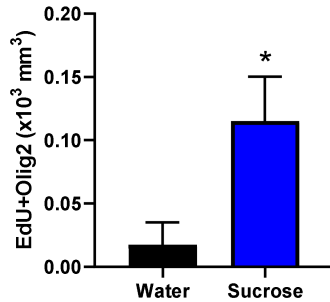**G**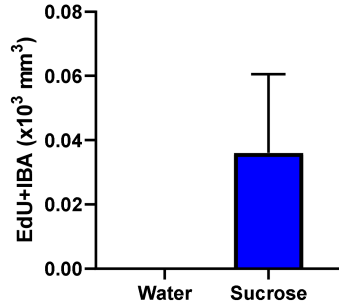

Supplement: Supplementary Figure 1 — Restricting access to sucrose consumption augments hippocampal oligodendrogenesis. (A,B) Restricted sugar consumption increased the density of EdU positive cells (green) in the dentate gyrus of the hippocampus. (C,D) There was no change in the density of stage 1 putative stem cells [EdU+/GFAP+, (C)] or stage 3 immature neurons [EdU+/DCX+, (D)] suggesting no change in neurogenesis, however, it appears that the increased density of EdU+ proliferative cells was mediated by an increased density of oligodendrocytes progenitors [EdU+ (green)/Olig+ (magenta), (E,F)]. (G) There was no difference in the density of proliferative microglia in the dentate gyrus, following restricted sugar consumption, compared to water controls. All images are colocalized with DAPI (blue). Representative image scale bar is 100 μm and close up representative image scale bar is 10 μm. [file Data_Sheet_1.PDF]
